# Non-stenosing Carotid Atherosclerosis and Arterial Stiffness in Embolic Stroke of Undetermined Source

**DOI:** 10.3389/fneur.2020.00725

**Published:** 2020-07-24

**Authors:** Maurizio Acampa, Pietro Enea Lazzerini, Chiara Manfredi, Francesca Guideri, Rossana Tassi, Carlo Domenichelli, Alessandra Cartocci, Giuseppe Martini

**Affiliations:** ^1^Stroke Unit, Department of Emergency-Urgency and Transplants, “Santa Maria Alle Scotte” General Hospital, Azienda Ospedaliera Universitaria Senese, Siena, Italy; ^2^Department of Medical Sciences, Surgery and Neurosciences, University of Siena, Siena, Italy; ^3^Department of Medical Biotechnology, University of Siena, Siena, Italy

**Keywords:** ischemic stroke, ESUS, carotid atherosclerosis, arterial stiffness, blood pressure, arterial hypertension

## Abstract

**Background and Purpose:** Recent findings suggested that non-stenosing atherosclerosis (NSA) may play an important pathogenic role, especially in cryptogenic strokes. Furthermore, arterial stiffness has been suggested to be a useful tool in identifying patients with embolic stroke of undetermined source (ESUS) with poor neurological prognosis. In this view, the aim of our study was to assess the association between carotid NSA and arterial stiffness in ESUS patients, in order to better define the cardiovascular risk profile of this subgroup of patients.

**Methods:** We enrolled 100 patients with ESUS (52 males, 48 females) and 48 patients with ischemic stroke from atherosclerosis. All patients underwent clinical and neuroimaging examination. A 24-h heart rate and blood pressure monitoring was performed in order to evaluate systolic, diastolic and mean blood pressure, pulse pressure, and arterial stiffness index (ASI).

**Results:** NSA was present in 48 patients. In comparison with non-NSA-ESUS, in NSA-ESUS the mean age was higher, neurological deficit was more severe, hypertension, and diabetes were more common; systolic blood pressure, pulse pressure, and ASI were higher. In particular NSA-ESUS had ASI levels similar to strokes due to atherosclerosis.

**Conclusions:** Our findings shed light on specific cardiovascular risk profiles underlying different subtypes of ESUS, suggesting the presence of increased arterial stiffness in NSA-ESUS patients with a risk factors profile similar to strokes due to atherosclerosis.

## Introduction

Embolic strokes of undetermined source (ESUS) represent one-third of ischemic strokes. Although embolism is presumably the underlying mechanism of these strokes, the current definition of ESUS seems to be too broad (1), including heterogeneous cases, such as occult cardiac conditions (atrial cardiopathy, patent foramen ovale), aortic arch plaques, or atherosclerosis of large cervical and intracranial arteries. Recent findings suggested that non-stenosing atherosclerosis (NSA) may play an important pathogenic role, especially in ESUS (2–4). According to such evidence, some ischemic strokes might be reclassified if high-risk non-stenosing plaque features were taken into account (5) and non-stenosing atherosclerosis could represent a specific phenotype separate from other subtypes such as from atrial cardiopathy (6).

It is well-known that arterial stiffness is associated with atherosclerotic burden and incident cardiovascular events (7) and may play a pathogenic role, especially in patients with large cerebral artery calcification, stenosis, or occlusion (8). A recent study showed that arterial stiffness during the acute phase of cryptogenic strokes might be useful in identifying patients at high risk of having a poor neurological prognosis (9), but no data are reported about the link between different subtypes of cryptogenic strokes and arterial stiffness.

In this view, the aim of our study was to assess the presence of the common cardiovascular risk factors and especially arterial stiffness, in patients with ESUS associated with non-stenosing extracranial/intracranial carotid atherosclerotic plaques, in order to define a more complete cardiovascular risk profile in this subgroup of patients.

## Materials and Methods

The data that support the findings of this study are available from the corresponding author upon reasonable request. We enrolled 100 patients, admitted consecutively to our Stroke Unit (Siena University Hospital) for ESUS, defined according to criteria proposed by the Cryptogenic Stroke/ESUS International Working Group (10). We also evaluated a confirmatory group composed of 48 patients with acute stroke from atherosclerosis. Neurological status at admission was assessed by using the National Institutes of Health Stroke Scale score (NIHSSs). All patients underwent neuroimaging examination (brain CT with angio-CT scan and/or brain magnetic resonance imaging), extracranial/transcranial arterial ultrasound, transthoracic echocardiography, 12-lead electrocardiogram, 24-h electrocardiogram. A 24-h blood pressure (BP) monitoring was conducted for all the subjects. The study was approved by the Ethics Committee of the University Hospital of Siena, Italy.

### Carotid Ultrasonography

All the patients underwent carotid ultrasonography procedure by means of iE33 ultrasound system (probe 1.8–3.6 MHz) (Philips Ultrasound, Bothell-Everett Highway, Bothell, WA, USA): in all patients ICA stenosis degree (ECST method) (11) and plaque characteristics were evaluated.

Plaques were classified according to the five type classification (12): Type 1 plaques were uniformly echolucent, type 2 predominantly echolucent, type 3 predominantly echogenic, type 4 uniformly echogenic, and type 5 consisted of plaques that could not be classified owing to heavy calcification and acoustic shadows.

### 24-h Blood Pressure Monitoring

BP monitoring was performed using validated oscillometric devices (Bedside Monitor Life Scope I BSM-2303K, International Div., Nihon Kohden Corp., Tokyo, Japan), in the first 24 h from the admission in the Hospital. BP was recorded for 24 h every 30 min, between 06:00 h to midnight, and hourly, from midnight to 06:00 h. For inclusion in the study, at least 80% of valid BP and heart rate measurements for each subject were needed.

The following parameters were evaluated: SBP, systolic blood pressure; DBP, diastolic blood pressure; MBP, mean blood pressure; PP, pulse pressure; and HR, heart rate.

The 24-h ABPM data were divided into daytime (06:00–22:00 h) and night-time (22:00–06:00 h) values. Dipping pattern was defined as follows: dippers had a ≥10% but <20% fall in SBP; non-dippers had an SBP fall <10% but >0%; extreme dippers had an SBP fall ≥20%; and reverse dippers had an SBP increase (13).

### Arterial Stiffness Index (ASI)

By plotting the individual values of SBP and DBP measurements, obtained through 24-h non-invasive monitoring, the linear regression slope of DBP on SBP was obtained and assumed as a global measure of arterial compliance, and its complement (1 minus the slope), named ASI, has been taken as a measure of arterial stiffness (14). Normal ASI values in older people were lower than 0.70 (15).

### Statistical Analysis

Statistical analysis was performed using the GraphPad Instat computer software (version 3.06 for Windows, GraphPad Software Inc., La Jolla, CA, USA) and R version 3.6.2. All results were presented as mean ± SD values. Normal distribution of quantitative variables was preliminary tested using the Kolmogorov–Smirnov test, instead, the homoscedasticity with Bartlett's test. Kruskal-Wallis or ANOVA tests, according to the normality distribution and the homoscedasticity, where used to compare mean differences of NSA groups with ATS confirmatory group; for qualitative variables, an approximation of Fisher exact test was used. When these tests were statistically significant *post-hoc* test were performed: Dunn's test for Kruskal-Wallis, multiple unpaired *t*-test with false discovery rate correction for ANOVA and multiple Fisher exact test with false discovery rate correction for approximation of Fisher exact test. Multivariate logistic regression was evaluated, and the stepwise procedure based on Akaike's criterion was performed. Odds Ratio (OR) and their confidence intervals (CI) were calculated. A *p*-value below 0.05 was considered statistically significant.

## Results

In our study, we enrolled 100 patients with ESUS. Table 1 presents demographic data concerning the study patients. Ipsilateral extracranial carotid NSA was found in 48 patients out of 100 patients with ESUS (Table 1); in this group carotid contralateral plaques were found in 38 patients. Table 2 shows the main characteristics of these plaques according to the five type classification; none of the patients presented carotid plaque ulceration and thrombosis.

**Table 1:** Clinical, hemodynamic, and laboratory parameters in ESUS with and without non-stenosing atherosclerosis and in stroke due to atherosclerosis.

	**Non-NSA-ESUS (*n* = 52)**	**NSA-ESUS (*n* = 48)**	**Stroke due to atherosclerosis (*n* = 48)**	***p*-value**
Age (years)	63 ± 14	77 ± 10	77 ± 9	**<0.001^a^^,^^b^**
Women/men	29:23	19:29	19:29	0.17
NIHSS score	10 ± 7	13 ± 9	13 ± 8	**0.033^b^**
Moderate-Severe stroke (NIHSSs ≥ 8)	23 (44%)	32 (67%)	34 (71%)	**0.02^a^^,^^b^**
Cardiovascular risk factors			
Hypertension, *n* (%)	30 (57%)	38 (79%)	38 (79%)	**0.03^a^^,^^b^**
Diabetes mellitus, *n* (%)	6 (11%)	15 (31%)	10 (21%)	0.06
Hypercholesterolemia, *n* (%)	25 (48%)	24 (50%)	29 (60%)	0.43
Previous CAD, *n* (%)	4 (7%)	5 (10%)	5 (10%)	0.9
Previous stroke/TIA, *n* (%)	4 (7%)	8 (17%)	8 (17%)	0.31
Smoking, *n* (%)	18 (35%)	15 (31%)	15 (31%)	0.93
Laboratory parameters			
Glucose (mg/dl)	115 ± 30	129 ± 41	134 ± 35	**0.005^b^**
Creatinine (mg/dl)	0.82 ± 0.3	0.93 ± 0.3	0.94 ± 0.3	0.12
Total cholesterol (mg/dl)	190 ± 42	185 ± 41	195 ± 32	0.50
LDL-cholesterol (mg/dl)	118 ± 38	114 ± 38	121 ± 29	0.62
HbA1c (%)	5.7 ± 0.6	6.2 ± 0.9	6.0 ± 0.7	**0.01^a^**
CRP (mg/dl)	0.9 ± 1.8	2.1 ± 4.0	3.1 ± 6.0	**0.05^b^**
Hemodynamic parameters			
SBP (mmHg)	130 ± 15	139 ± 17	141 ± 15	**<0.001^a^^,^^b^**
DBP (mmHg)	70 ± 9	71 ± 10	66 ± 8	**0.01^a^^,^^b^**
MBP (mmHg)	90 ± 11	93 ± 10	91 ± 10	0.230
PP (mmHg)	59 ± 11	67 ± 16	75 ± 15	**<0.001^a^^,^^b^^,^^c^**
HR (beats/min)	72 ± 11	73 ± 15	71 ± 12	0.85
ASI	0.61 ± 0.13	0.67 ± 0.13	0.70 ± 0.14	**0.006^a^^,^^b^**
Number of patients with arterial stiffness (ASI > 0.70)	14 (27%)	25 (52%)	25 (52%)	**0.012^a^^,^^b^**
Dipping pattern			
Dippers, *n* (%)	22 (42.3%)	10 (20.8%)	12 (25%)	0.16
Non-dippers, *n* (%)	21 (40.4%)	26 (54.2%)	27 (56.3%)	
Reverse-dippers, *n* (%)	9 (17.3%)	12 (25.0%)	9 (18.8%)	

*Data are expressed as mean ± SD. NSA, Non-stenosing atherosclerosis; CAD, coronary artery disease; SBP, systolic blood pressure; DBP, diastolic blood pressure; MBP, mean blood pressure; PP, pulse pressure; HR, heart rate; ASI, arterial stiffness index. Bold values are statistically significant (p < 0.05)*.

a *Non-NSA-ESUS was statistically different from NSA-ESUS*.

b *Non-NSA-ESUS was statistically different from group with atherosclerosis*.

c *NSA-ESUS was statistically different from group with atherosclerosis*.

**Table 2:** Location and characteristics of carotid plaques in ESUS with non-stenosing atherosclerosis.

	**NSA-ESUS (*n* = 48)**
Carotid ipsilateral plaque (n)	48 (100%)
Carotid stenosis degree (%)	39 ± 5
Plaque characteristics	
Type 1, *n* (%)	2 (4%)
Type 2, *n* (%)	14 (29%)
Type 3, *n* (%)	27 (57%)
Type 4, *n* (%)	2 (4%)
Type 5, *n* (%)	3 (6%)
Carotid contralateral plaques (n)	38 (79%)

As a confirmatory group we also evaluated a group of 48 patients with stroke due to atherosclerosis, comparable with NSA-ESUS in terms of age and sex (see Table 1).

NSA-ESUS patients and atherosclerotic stroke patients were older than non-NSA patients. The neurological deficit was more severe in patients with atherosclerotic stroke than in non-NSA patients. In both NSA-ESUS and atherosclerotic strokes, we found a substantially greater proportion of subjects with hypertension; furthermore, the laboratory parameters showed increased levels of HbA1c in NSA-ESUS in comparison with non-NSA group and increased levels of C-reactive protein (CRP) in strokes due to atherosclerosis in comparison with non-NSA group (Table 1).

### Hemodynamic Parameters

Considering the hemodynamic parameters, in NSA-ESUS, SBP, ASI, and PP were significantly higher than in non-NSA patients (Table 1). In comparison with strokes from atherosclerosis, NSA-ESUS had similar SBP, MBP, HR and ASI, but lower PP values and higher DBP values (Table 1). Considering the dipping pattern there were no significant differences among the three groups of patients.

### Multivariate Analysis

Multivariate logistic regression model was constructed to determine the factors associated with NSA-ESUS. The following variables were initially evaluated: age (>65 years), clinical severity of ischemic stroke (NIHSS score ≥8), presence of hypertension, presence of diabetes mellitus, C reactive protein levels, presence of arterial stiffness (ASI > 0.70). Akaike's information criterion showed that the best subset of associated variables for NSA-ESUS were the following: ASI > 0.70 (*OR* = 2.92 for ASI > 0.70, 95% CI 1.18–7.56), diabetes (*OR* = 3.66, 95% CI 1.21–12.34), age (*OR* = 5.03 for age >65 years, CI 1.94–14.18).

## Discussion

The novel findings of our study are the following: (1) ESUS patients with NSA have a specific cardiovascular profile risk, similar to patients with stroke due to atherosclerosis, (2) NSA-ESUS patients have higher ASI values in comparison with non-NSA ESUS.

Indeed, our results demonstrated that in ESUS patients arterial stiffness (ASI values >0.70) is significantly associated with the presence of non-stenosant carotid plaques, independent of other risk factors.

Arterial stiffness is a well-known risk factor for cardiovascular events (7), associated with worse cardiovascular outcomes and independent of traditional risk factors; in particular, in both ischemic and hemorrhagic strokes, high arterial stiffness index values have been observed (14, 16, 17), suggesting its prognostic and independent predictive value (14, 18).

The association between this subtype of ESUS and arterial stiffness is particularly important in order to explain some possible collateral effects of anticoagulant therapy in these patients. Indeed, in NSA-ESUS, a recent study showed that aspirin was safer than rivaroxaban, determining lower rates of major bleeding (19). Since previous studies showed that arterial stiffness may promote the occurrence of cerebral hemorrhage (17) and hemorrhagic transformation after ischemic stroke (14), it is possible that in NSA-ESUS this factor may favor hemorrhagic complications. In this view, these subtypes of ESUS are similar to strokes from atherosclerosis and probably antiplatelet therapy represents the most appropriate therapy (20).

Our cohort of patients presented higher NIHSS values in comparison with other previous studies (21, 22), even if we found lower NIHSSs values in non-NSA ESUS in comparison with patients with stroke due to atherosclerosis. It is possible that this discrepancy may be explained by the different characteristics of our cohort of patients, that is composed of ESUS patients admitted to our intensive care with more severe symptoms and, in most cases, after intravenous thrombolysis and/or mechanical thrombectomy. These more severe neurological deficits may be associated with arterial stiffness, as suggested by our previous study (23), because arterial stiffness could negatively affect collateral circulation development, damaging the structural intracerebral vasculature, thereby determining a greater size of ischemic lesion, and a consequent worse neurological deficit.

Lastly, our cohort of patients with non-NSA-ESUS had lower levels of CRP in comparison with strokes due to atherosclerosis; instead NSA-ESUS seem to have intermediate values, but not significantly different in comparison with both non-NSA ESUS and atherosclerotic strokes. It is well-known the pathogenic link among inflammation, atherosclerosis, and arterial stiffness (24), thus these intermediate values suggest a further similarity between NSA-ESUS and strokes due to atherosclerosis.

This study has the following limitations: the recruitment was mono-centric, the sample size is small, there are no data about the link between arterial stiffness and possible long-term complications (cardiovascular events, hemorrhagic complications). In this view our study represents a hypothesis-generating research, thus further studies are needed in order to confirm our findings.

## Conclusions

Our findings shed light on specific cardiovascular risk profiles underlying different subtypes of ESUS, suggesting the presence of increased arterial stiffness in NSA-ESUS patients with a risk factors profile similar to strokes due to atherosclerosis.

## Data Availability Statement

The raw data supporting the conclusions of this article will be made available by the authors, without undue reservation.

## Ethics Statement

The studies involving human participants were reviewed and approved by the Ethics Committee of the University Hospital of Siena, Italy. The patients/participants provided their written informed consent to participate in this study.

## Author Contributions

MA: conception and design of the work. MA, PL, CM, FG, RT, CD, and GM: substantial contributions to the acquisition of data for the work. MA, PL, CM, CD, and AC: substantial contributions to the analysis of data for the work. MA, PL, GM, and AC: substantial contributions to the interpretation of data for the work and revising the draft of the work critically for important intellectual content. MA, PL, and AC: drafting the work. MA, PL, CM, FG, RT, CD, GM, and AC: final approval of the version to be published and agreement to be accountable for all aspects of the work in ensuring that questions related to the accuracy or integrity of any part of the work are appropriately investigated and resolved. All authors contributed to the article and approved the submitted version.

### Conflict of Interest

The authors declare that the research was conducted in the absence of any commercial or financial relationships that could be construed as a potential conflict of interest.
